# Selective Down-Regulation of Nuclear Poly(ADP-Ribose) Glycohydrolase

**DOI:** 10.1371/journal.pone.0004896

**Published:** 2009-03-25

**Authors:** David M. Burns, Weihai Ying, Tiina M. Kauppinen, Keqing Zhu, Raymond A. Swanson

**Affiliations:** Department of Neurology, University of California at San Francisco and Veterans Affairs Medical Center, San Francisco, California, United States of America; Institut Pasteur Korea, Republic of Korea

## Abstract

**Background:**

The formation of ADP-ribose polymers on target proteins by poly(ADP-ribose) polymerases serves a variety of cell signaling functions. In addition, extensive activation of poly(ADP-ribose) polymerase-1 (PARP-1) is a dominant cause of cell death in ischemia-reperfusion, trauma, and other conditions. Poly(ADP-ribose) glycohydrolase (PARG) degrades the ADP-ribose polymers formed on acceptor proteins by PARP-1 and other PARP family members. PARG exists as multiple isoforms with differing subcellular localizations, but the functional significance of these isoforms is uncertain.

**Methods / Principal Findings:**

Primary mouse astrocytes were treated with an antisense phosphorodiamidate morpholino oligonucleotide (PMO) targeted to exon 1 of full-length PARG to suppress expression of this nuclear-specific PARG isoform. The antisense-treated cells showed down-regulation of both nuclear PARG immunoreactivity and nuclear PARG enzymatic activity, without significant alteration in cytoplasmic PARG activity. When treated with the genotoxic agent MNNG to induced PARP-1 activation, the antisense-treated cells showed a delayed rate of nuclear PAR degradation, reduced nuclear condensation, and reduced cell death.

**Conclusions/Significance:**

These results support a preferentially nuclear localization for full-length PARG, and suggest a key role for this isoform in the PARP-1 cell death pathway.

## Introduction

Poly(ADP-ribose) polymerases consume NAD^+^ to form poly(ADP-ribose) (abbreviated as PAR) on acceptor proteins. This post-translational modification influences protein-protein interactions and serves a variety of cell signaling functions [1]. PARP-1, the most abundant of the PARP family members, is localized to the cell nucleus and is activated by DNA damage. Activated PARP-1 forms PAR on histones, DNA repair enzymes, and proteins involved in gene transcription [1]. PARP-1 also forms PAR on PARP-1 itself, and this, under some conditions, can reduce PARP-1 activity [2]. Extensive activation of PARP-1 occurs after ischemia-reperfusion, trauma, and other conditions that cause DNA damage. In these settings, PARP-1 activation leads to NAD^+^ depletion, mitochondrial release of apoptosis-inducing factor, and cell death [3]–[5].

The PAR generated by PARP-1 and other PARP isoforms is degraded by poly(ADP-ribose) glycohydrolase (EC 3.2.1.143; PARG), for which there is only one identified genetic loci [6], [7]. The mitochondrial enzyme, ADP-ribosyl hydrolase, is also capable of degrading PAR [8], [9], but this is of uncertain biological significance because cells with PARG gene deletion exhibit massive PAR accumulation [6]. PARG activity can be suppressed by pharmacological inhibitors or RNAi, both of which slow the degradation of newly–formed PAR and block PARP-1 - mediated cell death [10]–[17]. However, PARG gene deletion is embryonic lethal in mice [6], and RNAi of the PARG orthologue in the nematode increases sensitivity to ionizing radiation [18]. Mice expressing a truncated PARG isoform lacking exons 2 and 3 in the regulatory domain show different effects on PARP-1 - mediated cell death in different experimental models [15], [19], [20].

**Figure 1:**
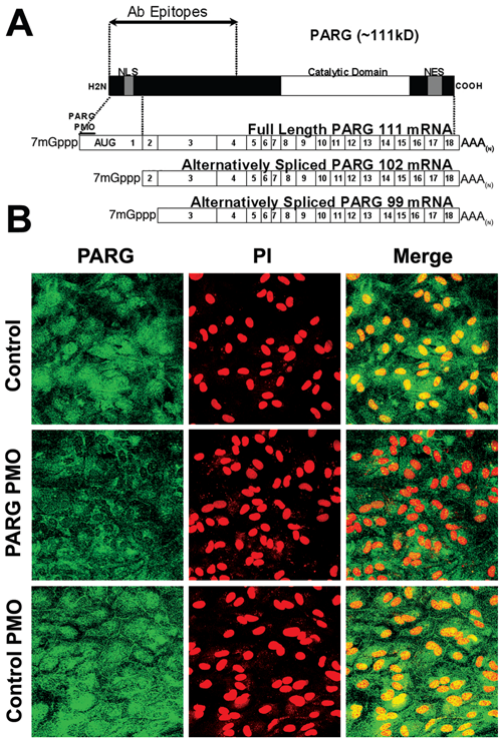
Nuclear PARG protein expression is reduced by the PARG antisense PMO targeted to exon 1. (A) The full-length 111 kD protein contains a nuclear localization sequence (NLS) in exon 1 and a nuclear export sequence (NES) near the carboxy terminus. The anti-PARG antibody targets the peptide fragment indicated [27]. The PARG antisense PMO was directed to the extreme 5′ end of exon 1 of the full-length PARG mRNA. (B) Astrocytes are immunostained for PARG (green), and nuclei are counterstained with propidium iodide (red). Merged images demonstrate a reduction in PARG expression, mainly in the nuclei, after PARG PMO treatment, as compared to both the no PMO (Control) condition and the inverted sequence PMO (Control PMO) condition. Scale bar = 20 µm. Images are representative of 6 independent experiments with similar results.

The interpretation of these somewhat conflicting observations is complicated by the existence of multiple PARG isoforms and the differing subcellular localizations of these isoforms. The human PARG gene can be processed into at least three splice variants (Fig. 1A). These splice variants yield proteins of 111 kD, 102 kD, and 99 kD. Of these, only the 111 kD isoform is preferentially localized to the nucleus [21]. However, several catalytically active smaller isoforms of PARG have been identified in cell lysates, and these account for the majority of endogenous PARG activity [7]. It remains uncertain whether these smaller species are splice variants or proteolytic fragments. Although full-length PARG can be demonstrated in cells transfected with PARG cDNA, endogenous expression of full-length PARG is not usually detectable, due either to low protein abundance or to rapid cleavage into smaller active fragments [7], [22]–[24].

In the present study we aimed to examine the specific role of nuclear PARG activity in PARP-1 – mediated cell death by selectively blocking translation of the 111 kDa, nuclear-targeted isoform using an antisense phosphorodiamidate morpholino oligonucleotide (PMO) targeted to the 5′ start of exon 1 (Fig. 1A). PARG enzymatic activity was used as the primary measure of antisense PMO efficacy in order to circumvent the difficulties inherent in identifying full-length PARG and active PARG cleavage products on standard Western blots [7]. Our findings indicate that selective down-regulation of the nuclear PARG isoform slows the rate of nuclear PAR degradation and attenuates PARP-1- mediated cell death.

## Materials and Methods

Reagents were purchased from Sigma Chemical Co (St. Louis, MO) except where noted. Animal studies were performed in accordance with a protocol approved by the San Francisco Veterans Affairs Medical Center animal use committee.

### Cell cultures

Primary mouse cortical astrocyte cultures were prepared by the method of Hertz [25] with minor modifications [5].

### Morpholino oligonucleotide treatments

The PARG antisense sequence, 5′-GGACCCGCAGCACACAGTCCCCC-3′, is complementary to the 5′ end of exon 1 on the full length PARG mRNA (GenBank, Accession# NM_011960), with the exception of the 3′nucleotide of the antisense sequence. The antisense sequence has no significant complementarity to other sequences in the NCBI Mouse RefSeq mRNA database. The inverted sequence also has no significant complementarity to other sequences in the NCBI Mouse RefSeq mRNA database, and was used as a negative control. For cell delivery, the PMOs (Gene Tools, Philomath, OR) were mixed with 1 µM ethoxylated polyethylenimine (EPEI) and diluted into the culture medium for a 3 hour incubation at 37°C. The cultures were used for experiments 2 days after PMO treatment.

### PARG activity assay

Exoglycosidic PARG activity was measured by the method of Menard and Poirier [26], with minor modifications [24]. The enzyme substrate, histone-bound ^14^C PAR, was prepared using calf thymus DNA and histones, [U-^14^C] NAD (2.84×10^−2^ pmol / dpm), and purified PARP-1 [24]. The PARG activity assay was preceded by a 1 hour incubation at 37°C to allow the digestion endogenous PAR. ^14^C-labeled PAR was added at a concentration of 5 µM and the incubation continued for an additional 30 minutes at 37°C. Aliquots were removed at the initial and ending time points, and reactions were quenched with 0.1% sodium dodecyl sulfate (SDS). All aliquots were also spiked with non-radiolabeled ADP-ribose for easy UV detection after thin-layer chromatography (TLC). The aliquots were spotted onto PEI-cellulose TLC sheets, which were developed first in 100% methanol and then in 0.3 M LiCl / 0.9 M acetic acid. The ADP-ribose spots were cut out and ^14^C was measured with a liquid scintillation counter. In some studies, the TLC sheets were imaged using the Typhoon 9410 system (Amersham) for quantification of ^14^C-ADP-ribose. Similar results obtained with the two methods. With either method, ADP-ribose formation was blocked by the PARG inhibitor gallotannin [10], [17] (not shown).

### Immunocytochemistry

Confluent astrocytes plated on glass coverslips were fixed in cold methanol / acetone. The cultures were incubated with a 1∶500 dilution of anti-N-terminal PARG polyclonal antibody (generous gift of Dr. Myron Jacobson) [24], [27], or a 1∶1000 dilution of rabbit anti-PAR (Trevigen), and visualized with fluorescent anti-rabbit or anti-goat IgG. For some studies, cell nuclei were counterstained with propidium iodide. Photomicrographs were obtained by confocal microscopy.

### Western blots

Cultures were lysed in modified RIPA buffer, applied to a 10% resolving SDS gel, separated by electrophoresis, and transferred to PVDF membranes as described [24]. The antibody dilutions were as follows: mouse monoclonal antibody to poly(ADP-ribose) (Trevigen , Gaithersburg, MD), 1∶500; rabbit polyclonal antibody to PARP-1 (Cell Signaling Technology), 1∶1500; mouse monoclonal antibody to porin (Calbiochem, San Diego, CA), 1∶1000; mouse monoclonal antibody to β-actin (Sigma-Aldrich), 1∶10,000. Primary antibodies were visualized with peroxidase-labeled anti-mouse or anti-rabbit IgG and chemiluminescence detection. Band intensities on each lane were normalized to the β-actin bands on the same lane. For quantification of poly(ADP-ribosyl)ated proteins, the signal over the entire lane (approximately 20–250 kD) was measured.

### Cell fractionation

The separation of nuclear and cytoplasmic components was performed as described [28]. The purity of the nuclear and cytoplasmic fractions of each separation were tested in duplicate using Western blots stained with antibodies to the nuclear marker, PARP-1, and the mitochondrial marker, porin.

### MNNG incubations and cell survival assays

MNNG incubations were performed in a balanced salt solution (BSS) containing (in mM): KCl, 3.1; NaCl, 134; CaCl_2_, 1.2; MgSO_4_, 1.2; KH_2_PO_4_, 0.25; NaHCO_3_, 15.7; HEPES, 5, glucose, 5. The BSS was equilibrated with a 5% CO_2_ atmosphere and the incubations were performed in a 5% CO_2_ 37° incubator. Incubations were terminated after 60 minutes, except where noted, by exchange with fresh BSS. Cell survival was evaluated 24 hours later by measuring lactate dehydrogenase (LDH) activity in cell lysates, using the method of Koh and Choi [29] with modifications [5]. Cell survival in each well was calculated by normalizing LDH activity to the mean LDH activity of 4 control wells from the same 24-well plate.

### Statistical analyses

Data are presented as means±standard errors. Statistical significance was assessed by using the analysis of variance (ANOVA) followed by the Student-Newman-Keuls test for comparisons between multiple treatment groups.

## Results

The antisense PARG PMO was designed to target the most 5′ AUG start codon, which is present only in the full-length 111 kD mouse PARG mRNA (Fig. 1A). Immunostaining for PARG in mouse astrocyte cultures treated with antisense PARG PMO showed a marked and selective loss of immunoreactivity from the nuclei of the antisense PMO-treated cells, as compared to sister cultures treated with control (invert sequence) PMO or no PMO (Fig. 1B). Controls prepared with omission of the anti-PARG antibody exhibited no immunoreactivity (not shown).

**Figure 2:**
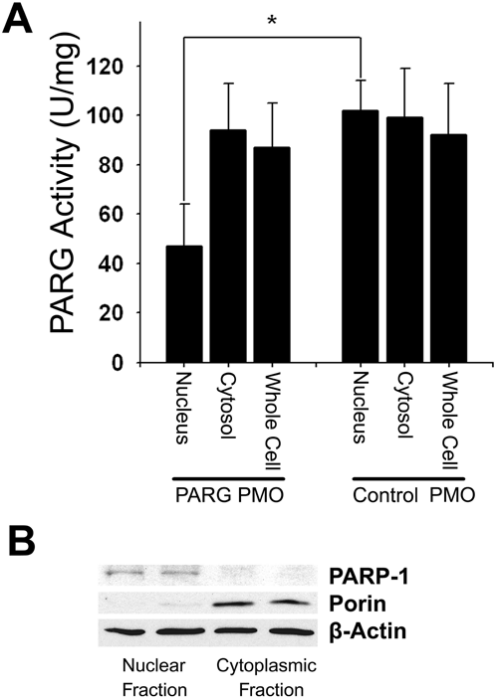
Nuclear PARG activity is selectively reduced by PARG antisense PMO. (A) Activity assays of whole-cell, nuclear, and cytoplasmic cell fractions. n = 5; * p<0.05. (B) Western blots of cell fractions showed the nuclear marker PARP-1 confined to the nuclear fraction and the mitochondrial marker porin confined to the cytoplasmic fraction. The two lanes show samples prepared from different culture plates. Blots are representative of 3 independent experiments.

The effect of antisense PARG PMO on full-length PARG expression was also evaluated with western blots of nuclear and cytoplasmic cell fractions; however, the western blots revealed only faint or undetectable full length PARG, consistent with prior reports of endogenous PARG expression [7], [22]–[24]. We therefore measured PARG enzymatic activity in the nuclear and cytoplasmic cell fractions to evaluate the functional effects of the antisense PARPG PMO treatment. As shown in Figure 2, the nuclear PARG specific activity (activity normalized to protein) was substantially lower in the cells treated with the antisense PMO than in cells treated with the control PMO or no PMO. By contrast, there was no significant difference in PARG cytoplasmic specific activity among the 3 treatment groups. As would be expected, there was no significant reduction in whole-cell PARG activity in the antisense-treated cultures, consistent with the relatively small contribution of nuclear protein to total cell protein and nuclear PARG activity to total cell PARG activity [7], [24].

**Figure 3:**
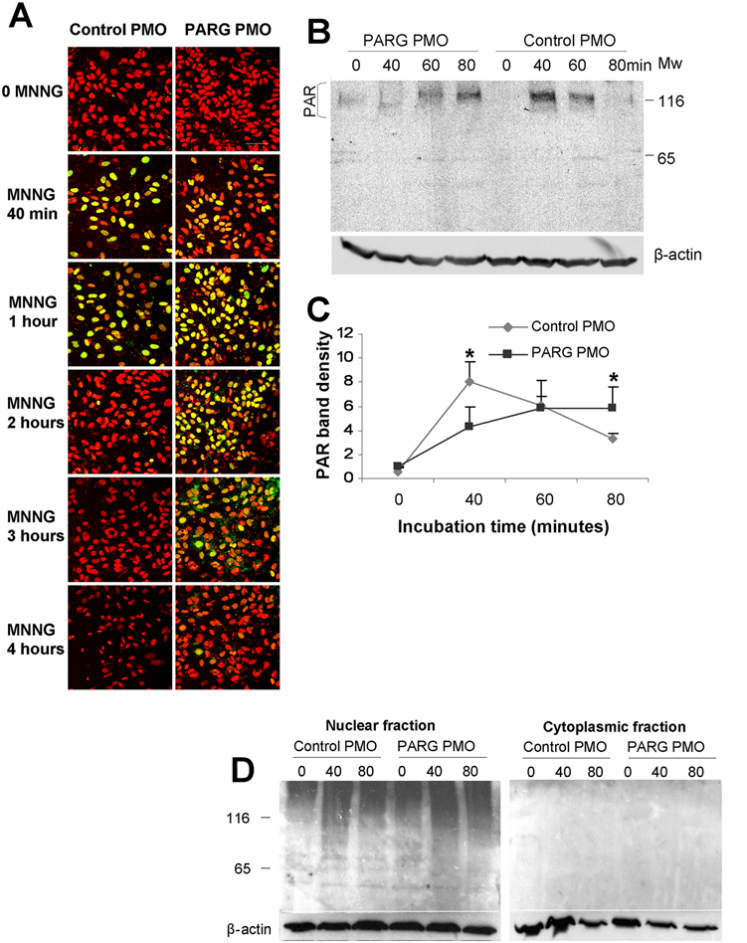
PARG antisense PMO slows PAR degradation. Cultures were incubated with 50 µM MNNG. For observations at time points of 60 minutes or less, incubation with MNNG was continuous. For observations at time points beyond 60 minutes, MNNG was washed out at the 60-minute time point. (A) Immunostaining for PAR (green) in astrocytes at the designated time points. Nuclei are counterstained red with propidium iodide, and the images are merged. The cultures treated with antisense PARG PMO show prolonged nuclear PAR immunostaining relative to those treated with control PMO, and show less nuclear condensation at the 4 hour time point. Scale bar = 40 µm. Results are representative of 4 independent experiments. (B) PAR Western blots from cells treated with MNNG show prolonged elevations in PAR immunoreactivity in the cultures treated with PARG antisense PMO. (C) Graph shows quantified data. n = 3, *p<0.05. (D) PAR Western blots prepared from nuclear and cytoplasmic cell fractions showed PAR detectable only in the nuclear fraction. These blots are overexposed, relative to the blots shown in Fig. 3B, to increase sensitivity.

PARG activity *in situ* was evaluated by assessing PAR levels at several time points after inducing PARP-1 activation with the DNA alkylating agent N-methyl-N′-nitro-N-nitrosoguanidine (MNNG). In cultures treated with control PMO, PAR immunoreactivity peaked at 40–60 minutes after MNNG incubation and fell to baseline within 2 hours. In cultures treated with antisense PARG PMO, PAR immunoreactivity peaked at about 60 minutes and remained detectable for up to 4 hours, consistent with diminished nuclear PARG activity (Fig. 3A). The PAR immunostaining was confined to the nucleus in both control and antisense-treated cultures. Of note, nuclear condensation was apparent at the 4 hour time point in cultures treated with control PMO, but not antisense PARG PMO.

PAR western blots were also used to evaluate the time course of PAR formation. Cells treated with MNNG showed a distinct band of PAR immunoreactivity at 116 kD, corresponding to PARP-1 auto-modification, along with less distinct labeling of proteins at a range of molecular weights, generally above 85 kD (Fig. 3B, D). This pattern is consistent with prior reports [5], [6]. Western blots prepared from cell lysates prepared at serial time points after MNNG exposure showed a time course of PAR formation and degradation pattern similar to that observed with immunostaining. These serial evaluations also showed the peak level of PAR formation to be lower in the cells treated with PARG antisense PMO than in the cells treated with control PMO (Fig. 3C). In agreement with the immunostaining results, western blots performed on nuclear and cytoplasmic cell fractions showed abundant PAR in the nuclear fractions but no detectable PAR in the cytoplasm of either the control or antisense PMO - treated cultures (Fig. 3D). These findings support the results obtained with the enzymatic assays indicating a reduction in nuclear, but not cytoplasmic PARG activity in the antisense-treated cultures.

**Figure 4:**
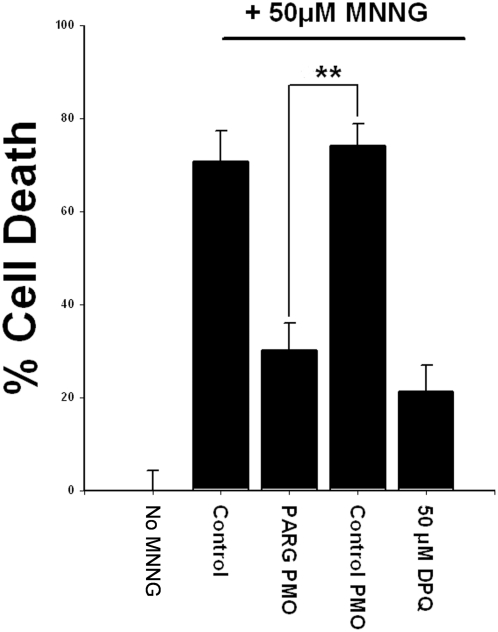
PARP-1 mediated cell death is reduced by PARG antisense PMO. Cell survival was assessed 24 hours after 60-minute incubations with 50 µM MNNG. Cell death was comparably reduced by co-incubation with the PARP inhibitor, DPQ. Data shown are representative of 5 experiments, each with n = 3. **p<0.01 vs. 50 µM MNNG alone.

Consistent with the reduced nuclear condensation observed in Fig. 3A, cells treated with PARG antisense PMO showed reduced cell death when evaluated 24 hours after MNNG incubations (Fig. 4). The magnitude of this effect was comparable to that achieved with the PARP inhibitor 3, 4-dihydro-5-[4-(1-piperidinyl)butoxy]-1(2H)-isoquinolinone (DPQ), suggesting a near-complete abrogation of PARP-1 – mediated cell death by the antisense PARG PMO.

## Discussion

Primary mouse astrocytes were treated with an antisense PMO targeted to exon 1 of full-length PARG to suppress expression of this nuclear-specific PARG isoform. The antisense-treated cells showed down-regulation of both nuclear PARG immunoreactivity and nuclear PARG enzymatic activity, without significant alteration in cytoplasmic PARG activity. When treated with the genotoxic agent MNNG to induced PARP-1 activation, the antisense-treated cells showed a delayed rate of nuclear PAR degradation, reduced nuclear condensation, and reduced cell death. These findings support prior reports that full-length PARG is preferentially localized to the nucleus [7], [21], and provide a novel tool for investigating potential isoform-specific functions of PARG. These results also suggest a key role for nuclear PARG activity in PARP-1 – mediated cell death.

The PARG gene can be processed into three splice variants that yield proteins of 111 kD, 102 kD, and 99 kD (Fig. 1A). Only the full length 111 kD PARG isoform is normally found in the cell nucleus, and this localization is attributed to a NLS motif encoded by the 5′ region of exon 1 in the full length PARG mRNA [7], [8], [21]. The selective reduction in nuclear PARG activity observed in cells treated with antisense PMO targeting the 5′ terminus of PARG exon 1 is thus consistent with a selective down-regulation of the full-length PARG isoform. However, evidence suggests that PARG normally found in the cytoplasm may under some conditions traffic into the nucleus [7], [30]. We thus cannot entirely exclude the possibility that the antisense treatment reduces expression of other PARG species with access to the nucleus, but the absence of any change in cytoplasmic PARG activity weighs against this possibility.

The morpholino PMO antisense approach used here to suppress nuclear PARG protein expression differs from siRNA and other antisense approaches in that it inhibits mRNA translation by a mechanism independent of mRNA destruction [31], [32]. Antisense PMOs have previously been used to selectively suppress translation of splice variant mRNA species, and evidence suggests that the PMO antisense approach has less off-target effects than siRNA and other antisense approaches [32], [33]. Nevertheless, the results presented here cannot entirely exclude the possibility the antisense PMO directed against the full-length PARG affects nuclear PARG activity by a mechanism independent of PARG protein expression, but the selective loss of nuclear PARG activity without loss of cytoplasmic PARG activity again weighs against this possibility.

Cellular PAR levels are the net result of the competing processes of PAR formation, primarily by PARP-1, and PAR degradation, by PARG. The prolonged increase in cellular PAR levels following MNNG-induced PARP-1 activation observed in cultures treated with antisense PARG PMO is consistent with reduced nuclear PARG activity. However, a somewhat surprising result was that the peak level of PAR formation was not elevated in these cultures, and was in fact reduced relative to the cultures treated with control PMO (Fig. 3C). Given that PARP-1 can be inhibited by PAR auto-modification [2], it is possible that the reduced peak PAR level observed in the PARG antisense-treated cultures is due to increased or prolonged PAR formation on PARP-1 with resultant reduced PARP-1 activity in these cultures. Reduced PARP-1 activity might also contribute to the reduced cell death observed in the antisense PARG-treated cultures. However, recent studies have also identified complex physical interactions between PARP-1, PARG, and other nuclear proteins [34], [35], and these findings raise the alternative possibilities that reduced nuclear PARG protein could influence PARP-1 activity by through these or other, more indirect interactions.

Confluent primary astrocytes cultures are a useful model for these studies because these cells are homogeneous, differentiated, slowly-dividing, and non-neoplastic, with a correspondingly low level of spontaneous apoptosis. Astrocytes are the most numerous cell type in mammalian brain. Prior studies have extensively characterized the PARP-1 - mediated cell death pathway in this cell type, and shown that PARP-1 - mediated cell death can be attenuated by pharmacological PARG inhibitors [5], [10], [11], [24], [36]–[38].

Results of the present study can be compared to recent reports in which PARG protein expression was reduced by other methods in other model systems. Ablation of PARG enzymatic activity by gene disruption in the mouse causes massive PAR accumulation and embryonic lethality [6]. Cortes et al. generated a viable mouse in which a truncated 60 kDa PARG enzyme was expressed in place of the full length 110 kDa enzyme as a result of the targeted deletion of the exons 2 and 3 in the regulatory domain [19]. Cells from these mice showed reduced PARG activity in the nuclei, but markedly increased activity in the mitochondrial fraction. Surprisingly, cells from these mice showed evidence of reduced, rather than increased auto-modification of PARP-1, possibly due to abnormal interaction between the truncated PARG and PARP-1 or XRCC1 [19], [20], [35]. The reduced PARP-1 auto-modification was associated with increased cell death and mortality under conditions that trigger PARP-1 induced cell death. Most germane to the present study is the siRNA knockdown of PARG in HeLa cells reported by Malanga and colleagues [16]. These authors achieved a roughly 85% reduction in PARG enzymatic activity in both the cytoplasmic and nuclear compartments. Cells with reduced PARG activity showed prolonged PAR immunoreactivity and reduced cell death after exposure to H_2_O_2_, similar to the findings reported here with selective down-regulation of nuclear PARG activity. However, in contrast to the present results, PARG down-regulation did not increase viability in response to MNNG, a difference that may be attributable to PARP-1 – independent cell death pathways that can be triggered in dividing tumor cells by low concentrations of MNNG [39] or to the reduction in cytoplasmic PARG activity in the siRNA study.

The present results indicate that selective reduction of nuclear PARG activity can attenuate PARP-1-induced astrocyte death, but the mechanism of this effect remains to be established. Given what is known about PARP-1 cell death pathway, possibilities include 1) slowed PAR liberation from the nucleus [4] ; 2) slowed NAD^+^ consumption, due to reducing PAR turnover at acceptor sites or to increased PARP-1 auto-modification [5]; and 3) effects on PAR-regulated transcription factors [40]. Further work will be required to distinguish between these or other possibilities.
